# The Practice of Birth-Control

**Published:** 1921-05-21

**Authors:** 


### THE EDITOR'S LETTER-BOX.

**Correspondence on all subjects is invited, but we cannot in any way be responsible for the opinions expressed by our correspondents, who must give their name and address as a guarantee of good faith, but not necessarily for publication. Correspondents are reminded that brevity of style and conciseness of statement greatly facilitate early insertion.**

### The Practice of Birth-Control.

*To the Editor of* THE HOSPITAL.

SIR,—Concerning the discussion in your paper on "The Practice of Birth-Control," do certain big considerations simply not count at all? Such considerations as fair play to the unborn, the right to life, the immortality of the human soul, and the will of God?

I am one of ten. We are all alive—very much so—and having lapped up most of our father's worldly substance in food, clothing, education, and professional equipment, etc., all those among us who are old enough are busily engaged in merrily paddling our own canoes. Our mother (herself one of thirteen) did not appear to think any of us superfluous, neither does she now seem at all "exhausted," being quite energetic, happy, and pretty. In our home the doctor practically never entered except when one or other of us was coming into the world.

"Life is checkered shade and sunshine"; but for all that I am glad that I came into the world; I am glad that I have an immortal soul so that I shall never have to sink back into nothingness. My life is very precious to me and I am greatly enjoying it; but so is my youngest sister away at school enjoying hers, yet had my parents, for monetary or any other considerations, decided that five—six—seven—eight or nine children were as much as they could do with, then would this youngest little sister of mine not be existing to-day—and she has such a joy in life! Have I, and those older than I, more right to live than she has? Or can any of us in our various callings declare any other one among us to be unnecessary? Do we grudge each other? Do any of us believe that God really did not care whether we came into existence or not? And if He did not mean us all to be here, could He not have stopped us coming Himself, or taken some of us away again? Is it not just possible that He created the ten of us to carry out ten different objects?

Has Dr. Marie Stopes any right to decide how many souls may be given a chance of a useful life in this world and a happy one in the next? She has secured a place for herself in the scheme of things, but would debar others from their share.

It seems that these clinics of hers will come into being and prosper. If we cannot prevent death why then let us prevent life, and let the "waste spaces" of the earth continue to give their beauty for the beasts, birds, fishes, and insects to enjoy, and let Heaven be emptier than God originally intended it to be, because once upon a time He made people who developed into "birth controllers," because He made them and gave to them the gift of free will which He gives to us all, and because before they were born nobody had the brilliant idea of "controlling" them.

RADIOGRAPHER.

North Stafford Infirmary,
Hartshill, Stoke-on-Trent.

